# A Proposal for Clinical Genetics (Genetics in Medicine) Education for Medical Technologists and Other Health Professionals in Japan

**DOI:** 10.3389/fpubh.2014.00128

**Published:** 2014-08-25

**Authors:** Hidetsugu Kohzaki

**Affiliations:** ^1^Department of Cell Biology, Institute for Virus Research, Kyoto University, Kyoto, Japan; ^2^Department of Molecular Medicine, Graduate School of Medicine, Osaka University, Suita, Japan; ^3^Faculty of Allied Health Science, Yamato University, Suita, Japan

**Keywords:** genetic and chromosome testing, human and medical genetics, education, medical technologist, health professionals

## Abstract

Since the completion of the Human Genome Project, technology has developed markedly in fields such as medical genetics and genetic counseling in the medical arena. In particular, this technology has advanced the discovery of and ways of understanding various genes responsible for genetic diseases, and genetic polymorphisms thought to be associated with disease. Some have been implicated as factors in common lifestyle diseases and have increased the significance of genetic testing. In Japan, doctors and other health professionals, such as nurse and medical technologists have been engaged in genetic testing and genetic disease treatment. Chromosomal and gene aberrations were detected mainly by medical technologists. However, due to the nature of medical technologists who have to provide various clinical tests, such as blood test, pre-medical technology students are required to cover tremendous knowledge of different academic fields to pass the national exam. Therefore, the time allowed for such students to study chromosomal and gene analysis is quite limited. Moreover, they are forced to enter the medical setting without receiving sufficient training. Among them, only few medical technologists specialize in chromosomal and gene analysis. However, with the advancement of clinical genetics and development of chromosomal and gene analysis, conducting clinical practice is becoming more and more difficult for medical technologists who just passed the national exam. Also, doctors and other health professionals have not been able to keep up with service demands either. This paper attempts to address knowledge and skills gaps (especially clinical genetics, English, and ICT literacy) of medical technologists and we propose educational methods to prepare medical genetics professionals in Japan to meet these gaps.

## Introduction

The development of human and medical genetics education is associated with advances in molecular biology, immunology, and cancer research. In Japan, an interest in Mendelian genetics^1^ developed very early for the purpose of breeding, especially of plants. After World War II, the study of radiation genetics^2^ and human cytogenetics^3^ grew in the wake of the atomic bomb disasters at Hiroshima and Nagasaki, and research has continued to be pursued. But medical and human genetics education had been particularly weak (1). The Japanese were previously ashamed of having a handicapped child and they tended to be pessimistic about the child’s future. Prior to enactment of the Eugenic Protection Act in 1948, patients with “mental retardation,” an “anomaly,” or “leprosy” were prohibited from having children, and their sterilization was lawfully performed. Except for medical doctors, there was an absence of medical and human genetics education until recently. Though medical genetics training is required in the US for medical doctors, there is no such requirement in Japan; therefore, at many Japanese medical institutes, there are very few medical and human genetics specialists.

## Genetic Testing in Japan

Prior to the establishment of genetic counseling in Japan, chromosomal and gene aberrations were detected mainly by medical technologists. Their numbers, however, are insufficient to meet current demand in Japan. As a result, members of a variety of other professions are eligible for certification to engage in chromosome and gene testing, including physicians, researchers, and various paramedics (Table 1 contains a list of professions that may be certified). The certifying bodies of service providers of genetic counseling vary in Japan: “clinical cytogeneticist,” Japanese board of medical genetics (JSHG) (2); “genome medical research coordinator (GMRC) (2),” JSHG; “genetic medical technologist,” Japanese association for chromosome and gene analysis (JACGA) (3), and “molecular analysis technologist,” College of Molecular Analysis of Japan (CMAJ) (4). Table 1 contains a description of the various professionals and their activities with respect to genetic testing and/or counseling.

**Table 1:** **Professions certified to engage in chromosome and gene testing and/or genetic counseling in Japan**.

Profession*	Nature and tasks	Accreditation organization
Genetic counselor^a^ (in Japan) (5)	Genetic counselors are required to not only provide genetic medical information but also assist patients in managing the psychosocial implications of their genetic risks and/or genetic condition	The Japan Society for Genetic Counseling (5)
Clinical cytogeneticist^b^	A certification system was initiated for physicians, researchers, and technologists engaged in chromosome testing. Since chromosome testing is directly related to medical care, including prenatal diagnosis in many cases, certified clinical cytogeneticists use advanced testing techniques and provide patients with accurate, relevant information necessary for medical care	The Japanese Board of Medical Genetics in the JSHG (2)
Genome medical research coordinator^c^ (GMRC)	To apply and use genome medical research findings for actual medical practice and health promotion, participation by many collaborators for research is essential. Collaboration by persons who assist in obtaining informed consent (IC) with sufficient ethical consideration is essential for studies on diseases with a certain level of incidence. The JSGH designated the person that assists in obtaining IC as a GMRC and established a certified education system. This system guarantees GMRC quality and facilitates the acquisition of knowledge in rapidly progressing genome medical research	The JSHG (2)
Genetic medical technologist^d^	The JACGA’s goal is the appropriate use of clinical chromosome and gene testing and maximum feedback about test results, by preparing technologists with specialized knowledge and skills regarding advanced techniques and gene testing. JACGA is established mainly by medical doctors, medical technologists, and scientists to improve and spread chromosome and gene testing techniques. Objectives of this profession include promoting development and expansion of chromosome and gene testing, securing the safety of medical care, and reassuring patients about the accuracy of testing, thus, contributing to improved national medical care	The JACGA (3)
Molecular analysis technologist^e^	To develop and expand gene analysis and its related tests through the nurture of molecular analysis technologists with the advanced technical knowledge and skills of gene analysis. Objectives of this profession also include promoting improvement and standardization of technical levels of gene analysis, and reassuring patients about the accuracy of testing, thus, contributing to the public’s health and development of science technology. This profession consists of two levels: primary and first levels	The CMAJ (4)
Other health professionals^f^	Paramedics include medical technologists and nurses	Japan government

*^a–e^Private qualification. Job contents of the professions b, d, and e are almost the same. There is an excess number of chromosome and gene analysis institutions in Japan. Medical technologists wishing to become profession “a” require a postgraduate degree*.

*^f^ National qualification. This profession is distinctive from other professions. Examination subjects required for this profession are also different from those required for other professions*.

*These professions were established in order to support medical technologists conducting chromosomal and gene analysis with specialized knowledge and skills.

These service providers have received the requisite education and passed examinations for certification. Thus, physicians, researchers, and medical technologists/other health professionals working in hospitals have been screened and educated; nevertheless, the depth and breadth of their education are limited due to time constraints. Most are not sufficiently educated with respect to clinical genetics (genetics in medicine), and therefore, many may not fully recognize its importance. This is clear when their educational preparation (“genetic literacy”) is compared to the genetics education described in a report from the ASHG Information and Education Committee (5).

In Japan, it is thought that any health care worker can perform chromosome and genetic testing. Thus, it appears that medical technologists are responsible for some chromosome and gene analysis, with other health professionals responsible for the remainder. We would like to recommend the implementation of legal regulations or guidelines that specify the knowledge and skills necessary to engage in chromosome and genetic testing.

## Educational Needs of Genetics Professionals in Japan

In this section, the educational methods to train next-generation students/other health professionals belonging to medical care-associated educational institutions in Japan are discussed. The content is based primarily on our previous research and education regarding genetics. Currently, we are engaged in chromosome and gene analysis/gene testing education based on content assessed by the National Examination for Medical Technologists in Japan. The areas are discussed below.

Practical components include chemistry, biology, biochemistry, physiology, anatomy, and hematology for not only medical technologists/other health professionals but also most physician Ph.D. medical geneticists (5–7) on chromosome and gene analysis/gene testing education in the third year of a 3-year educational program after completion of corresponding lectures. Lectures primarily involve genetics, molecular biology, and clinical genetics. Practical components include investigation of the polymorphism^4^ of aldehyde dehydrogenase (ADH2) (8, 9) in each student himself or herself. The objective is to allow students at an age when they begin drinking alcohol to compare their own alcohol resistance (phenotype) between ADH2 and SNP^5^ and to actually realize changes in a single gene and subsequent differences in the phenotype. Alcohol resistance and the effect of anesthetics are considered to be correlated, which also suggests that this material is appropriate for students who are going to be engaged in medical care. Students’ alcohol consumption, as a phenotype, is compared with their genotype. Each student reviews whether or not there is a correlation between gene polymorphism and alcohol consumption (phenotype), and submits a report.

The lecture contents include the basics of molecular biology and experimental procedures such as the PCR method^6^; this method is applied for the detection of hereditary diseases, such as neurological and metabolic/endocrine disorders, infection with pathogenic bacteria/viruses, and cancer, as well as for individual identification. Chromosomal abnormalities are covered by lectures on hematology (10), but a thorough understanding is required for the gene test education program. Currently, time is restricted. To aid understanding, the above practical genetic training about ADH2 and SNP is carried out despite the tightly scheduled curriculum.

To confirm the usefulness of this training for the understanding of gene tests as an example of our attempts, the results of examinations regarding gene tests between the two groups with and without such training were compared. The lecture contents and examination questions were matched between the two groups. The mean scores for students with (*n* = 179) and without (*n* = 102) training were 76.4 and 75.6, respectively (perfect score: 100). A *t*-test yielded no significant between-group differences (11). This was possibly because the examination questions covered basic content, and not practical content. In the future, we are going to see how these outcomes are affected on the national examination.

To confirm whether or not chromosome and gene analysis/gene testing education contributes to the rate at which students pass the National Examination for Medical Technologists in Japan, questions in national examinations over the past 6 years were investigated, and the proportion of students who gave correct answers to gene test-matched questions versus biochemistry/information science-matched questions was compared. The mean score for the field of chromosome and gene analysis/gene testing was more than 10 points lower, reducing the rate at which students passed the National Examination for Medical Technologists (see Table 2 for a summary of scores in the last 6 years).

**Table 2:** **Test scores on the national examination for medical technologists in Japan**.

NE	Genetic testing		Biology and biochemistry		Information science		National examination (NE)					
	KCHH	NE	KCHH	NE	KCHH	NE	KCHH		Ministry of health, labor, and welfare			
	Average	Number	Average	Number	Average	Number	Average	Pass rate	Pass rate	Examinees	SCI	Number
56th	47.8	5	75.8	16	70.3	4	66	78.7	67.8	4,060	2,753	200
55th	69.5	2	70.7	17	69.5	4	68.6	91.8	71.8	3,701	2,657	200
54th	64.7	3	68.2	19	48.7	3	65.2	81.6	73.7	3,997	2,947	200
53rd	73	6	77	8	65	4	65.3	80.5	74.7	4,023	3,004	200
52nd	59.5	6	87.3	15	77.3	3	70.8	94.9	72.9	4,071	2,968	200
51st	51.8	4	68	15	70.8	4	67.5	85.5	75.2	4,025	3,164	200
Average	61.05	4.3	74.5	15	66.9	3.7	67.2	85.5	72.7	3979.5	2915.5	200
SD	4.04		2.99		3.67		0.9	2.67	1.1			
*N*	6	26	6	90	6	22	6	6	6	23877	17493	1200

*KCHH, Kyoto College of Health and Hygiene. SCI, total number of Japanese medical students who passed the exam. Approximately 4,000 students apply for the exam every year, and recent examination passing rates remain around 70%. NE takes place only once in a year. The exam consists of 200 questions, and 60% accuracy is required to pass the exam. If failed, applicants must wait another year. Applicants’ correct choice to each question of the above subjects will change their lives*.

For training, basic procedures, such as genome DNA collection, agarose gel electrophoresis^7^, PCR, and Southern hybridization^8^, are selected (12). Table 2 would show genetic training increases the passage rate on the national examination. There may be a gap between these procedures and medical technologists’ work that are not being addressed by current standards in Japan. We plan to invite medical technologists engaged in viral, chlamydia, and tubercle bacillus testing in clinical practice to give lectures. In the future, the effects of these lectures will be examined. Nevertheless, it may be difficult for medical technologists to acquire knowledge regarding chromogene testing at the same level as required for the authorized genetic counselor-training course in Japan (5).

Medical technologists accrue this experience in clinical practice; this is inconsistent with the authorized genetic counselor-training curriculum. Therefore, in order to be allowed to provide clinical genetics services, they should be required to acquire one of the credentials described in Table 1. (Genetic counselors are required not only to obtain a postgraduate degree but also to have intensive training in genetics, psychological techniques, and ethical reasoning.) We have thought eligibility requirements are exacerbating the shortage of higher qualified professionals.

However, individuals do not always meet eligibility criteria for taking the examination. There are regulations that restrict eligibility for this examination using a point system according to whether an individual wrote papers during a limited time period, presented reports at conferences of designated scientific societies, and participated in conferences and training (periodic renewal is also necessary). For some qualifications, it is necessary to engage in training under senior instructors for some years (especially, clinical cytogeneticist). Therefore, eligibility requirements cannot be readily obtained.

It may therefore be necessary to establish specialized programs and institutions to prepare individuals to acquire these qualifications.

## Discussion

### Recommendations for “genetic literacy” education

We have been engaged in education regarding chromosome and gene analysis/gene testing (5, 6). Human genetics is not taught in Japanese junior high or high schools (5). However, the spirit of the Maternal Protection Act, bioethics, and famous genetic diseases associated with chromosomal abnormalities should be taught during compulsory education. We consider that students understand the importance and risks of chromosomal defects/genes. The results of examinations regarding gene tests have been favorable. However, they are not reflected in the results of national examinations. Although equipment is limited, a curriculum involving practice programs regarding viruses and pathogenic bacteria must be established. It is necessary to train medical technologists who can pass the national examination and demonstrate their ability in clinical practice. In actuality, genes associated with diabetes, stroke, heart disease, hyperlipidemia, hypertension, and obesity have been identified (OMIM) (13). In the future, chromosome and gene analysis/gene testing may be introduced as a screening method managed by medical technologists. According to WHO guidelines, other health professionals should work in the medical genetic fields.

Chromosome and gene analysis/gene testing have become more common, as described previously, but they involve obtaining important personal information, and persons engaged in this service provision are required to have precise and accurate knowledge and skills (14–23). Educational content and methods must keep pace with advances in genetics. Intensive training in genetics, psychological techniques, and ethical reasoning is needed to prepare medical technologists (and other healthcare professionals) to provide appropriate genetic counseling services. Both classroom/laboratory methods and supervised clinical experiences are needed. Information on effective educational methods in guidelines developed by the American Society of Human Genetics and in curricula regarding clinical genetics (genetics in medicine) in the United States (24) should be provided.

Recently, the position of genetic nurse has been established in the UK (5), and training for this position has begun in Japan. According to WHO guidelines, not only physicians but also all health care providers, such as nurse sand public health nurses, are advised to have the ability to provide genetic counseling irrespective of their professions. According to WHO, the profession and population ratio of medical geneticists in the advanced countries is about 1:220,000, and 1:3,700,000 in developing countries and Japan. Therefore, Japan needs to accelerate further training (25, 26).

### Our proposals

With the advancement of clinical genetics and development of chromosomal and gene analysis, conducting clinical practice is becoming more and more difficult for medical technologists who just passed the national exam. Chromosomal and gene analysis services require medical technologists/other health professionals to be current with respect to new genetic information and technology. The credentials described in Table 2 must be voluntarily acquired, so that will not only facilitate them with knowledge and skills but also reassure patients about the accuracy of testing.

First, the basic medical science taught during the compulsory education must be reacknowledged. Second, we must improve ICT literacy skills to keep ourselves updated with rapid advancement of medical devices and ICT (5). English literacy skills also must be acquired in order to be current with cutting edge clinical genetics in this global society (5). Finally, for professions with high turnover rates, such as nurses or medical technologists, and medical providers who left their career for raising their children, a training program should be prepared to help them return to work.

## Conclusion

In order to solve the above problems, together with physicians, medical geneticists, and medical technologists, other than the credentials described in Table 1, we plan to establish a new society and a national qualification system for “genome consultants” (or “genome inspectors”) (tentative name) in order to put them on equal footing with physicians and provide patients with accurate information on chromosome and genetic testing (5). In addition, genome consultants could encourage younger generations to have an interest in genetic counseling. We hope to instill unified high level of knowledge and skills via a master’s degree as Genetic counselors and the new national qualification system. We also hope that genome consultants will work not only in Japan but all over the world.

We also hope that these professions will contribute to genomic medicine, while delivering our advanced healthcare techniques not only in Japan but all over the world.

## Conflict of Interest Statement

The author declares that the research was conducted in the absence of any commercial or financial relationships that could be construed as a potential conflict of interest.

## Footnotes

^1^Mendelian genetics: Mendelian inheritance was initially derived from the work of Gregor Johann Mendel published in 1865 and 1866, which was re-discovered in 1900. It was initially very controversial. When Mendel’s theories were integrated with the chromosome theory of inheritance by Thomas Hunt Morgan in 1915, they became the core of classical genetics. His law consists of three tree parts. First: the Law of Segregation, second: the Law of Independent Assortment, and third: the Law of Dominance.

^2^The genetic effects of radiation are investigated.

^3^Mechanisms for genetic phenomena are investigated by comparing cytological findings, such as chromosomal behaviors, polyploidy, and aneuploidy during cell division, with the laws of inheritance. Chromosomal behaviors according to Mendel’s laws and mechanisms for sex determination have been cytogenetically elucidated.

^4^Several genotypes exist for a certain gene.

^5^Individual differences in DNA nucleotide sequences are called genetic polymorphisms. In SNP, only a single base is replaced by another. About 10 million SNPs exist in the 3 billion bases of the human genome. Some SNPs in gene regions influence protein expressions.

^6^Trace amounts of DNA are amplified using polymerase, an enzyme involved in DNA replication.

^7^Negatively charged DNA molecules are separated by agarose pores according to their size.

^8^Two single-stranded DNA molecules complementary to each other form a double-stranded DNA molecule through hydrogen bonds formed between bases. The size of DNA molecule of interest can be determined by agarose gel separation.
